# Supplementary material for: A novel few-shot object detection framework for multi-scene driving based on contrastive proposal encoding
Source: PLoS One. 2025 Dec 30;20(12):e0337541. doi: 10.1371/journal.pone.0337541 (PMC12752960; doi:10.1371/journal.pone.0337541)
Supplement: Attachment — Submitted filename: Response_to_Reviewers.pdf [file pone.0337541.s002.pdf]

Response to Reviewers

Title: A Novel Few-Shot Object Detection Framework for Multi-Scene Driving Based on Contrastive Proposal Encoding

Manuscript Reference Number:
PONE-D-25-27923

Authors:
Yalei Dong
Jing Xiao
Fengchen Wei

Date: October 01, 2025

Message from the Authors

Dear Editors and Reviewers,

We thank you for your constructive comments, which have allowed us to improve the quality of the manuscript. We have addressed the comments and incorporated your valuable suggestions in the revised manuscript, in particular highlighting the key contributions of this work..

We address each comment separately in the following detailed response. The comments we received are boxed, and our responses are written following each comment. We look forward to hearing from you and hope that you find the revised manuscript satisfactory.

Sincerely,
Yalei Dong, Jing Xiao, Fengchen Wei

Response To Editor

Overall Comments

1.Before starting the methodology, it would be better to provide summary of related works including Author, year, dataset, models, best model, feature selections techniques, accuracy, and processing time ....et. because the related works did not clearly indicate the research gaps.

2.Before starting the methodology, it would be better to provide a summary of related works, including author, year, dataset, models, best model, feature selection techniques, accuracy, and processing time, as the related works did not clearly indicate the research gaps. Additionally, your proposal is based on ref.[15] largely. However, the reference [15] is often not the dominant one for improving your method. Therefore, to enhance the manuscript, consider incorporating more recent advances and relevant research works.

3.All detection algorithms rely on underlying mathematical principles. The absence of a mathematical framework in your paper renders your method insufficient. Without using a mathematical equations, the methodology section is still weak and unclear.

4.There is a lack of description of Fig. 1 and Fig. 2. Please add a brief description- a short explanation of what the figure illustrates.

5.There are a lot of different classifier methods. The paper includes only a Softmax classifier to design an ensemble model. Why? Did you try other classifiers when conducting a set of your experiments or were you depending on previous studies? Authors should discuss this point in the discussion section to be clarified in their manuscript so that their model can actually be supported.

6.The quality of a manuscript's results section is significantly improved by the use of well-designed, clear, and appropriate figures and tables that effectively present complex data, summarize key findings, and help readers visualize trends and significant information without unnecessary text. However, in reviewing your manuscript, I noticed that your manuscript's results just included tables without figures, which may limit the clarity of your findings and the chance of acceptance of the manuscript for publication.

7. In reviewing the bibliography, I noticed there is a repetition of the same reference [15, 17] in the bibliography. It should be corrected.

8.In the abstract, the Future work is unclear. It needs more explanation to guide the reader on the next steps.

Response

We would like to thank you for collecting and relaying the review responses to us. We are resubmitting the revised manuscript with the aim to highlight the contributions of this work.

Response To Reviewer

Reviewer Comment

> 1.Before starting the methodology, it would be better to provide summary of related works including Author, year, dataset, models, best model, feature selections techniques, accuracy, and processing time . . . .et. because the related works did not clearly indicate the research gaps.

Response

In accordance with your suggestions, we have systematically sorted out and re-edited the ”Related Work” section Firstly, the existing research is presented in a logical framework of ”Author, year, dataset, models, best mode”, and at the same time, the core performance indicators (including accuracy) and model processing time of each study are supplemented, making the comparison dimensions of different studies more complete and the information more intuitive. Secondly, a dedicated paragraph has been added at the end of this section. By combining the existing research progress sorted out in the previous text, it clearly points out the key issues that have not been resolved in the current field. Specifically, these include that most of the existing methods are verified based on general datasets and have not been targeted at multiple scenarios in vehicle detection. Although multi-scale attention has been introduced, the adaptation mechanism has not been designed in combination with the target scale fluctuations in different scenarios of vehicle detection. This lays a clearer logical foundation for the subsequent proposal of the research method in this study. The revised ”Related Work” section not only enhances the completeness of the literature review but also strengthens the innovation and necessity of this study by clarifying the research gaps. We hope it can meet your requirements.

Reviewer Comment

> Before starting the methodology, it would be better to provide a summary of related works, including author, year, dataset, models, best model, feature selection techniques, accuracy, and processing time, as the related works did not clearly indicate the research gaps. Additionally, your proposal is based on ref.[15] largely. However, the reference [15] is often not the dominant one for improving your method. Therefore, to enhance the manuscript, consider incorporating more recent advances and relevant research works.

Response

Regarding the opinion of ”supplementing relevant work summaries before the methodology begins and clarifying research gaps” : We have systematically improved the ”Related Work” section and strictly followed the logical framework of ”author - Publication year - Dataset used - Model architecture - Best model selection - feature selection technique” to sort out and summarize the existing research. At the same time, the key performance indicators (including accuracy) and model processing time of each study were supplemented, making the comparison dimensions of

different studies more comprehensive and the information transmission clearer. Based on this, we have added a dedicated paragraph at the end of ”Related Work”, which, in combination with the previous review content, clearly points out the research gaps existing in the current field, laying a clear logical foundation for the subsequent proposal of the methodology in this study.

Opinions on ”supplementing the latest progress and related research, and optimizing the citation of references” : We attach great importance to this suggestion. By searching for the achievements of top journals and conferences in the field over the past three years, we have added several latest related studies focusing on this research direction and included them in the ”Related Work” section for comparative analysis. This not only supplements the cutting-edge trends in the field but also further highlights the innovative value and differentiated advantages of this research. Meanwhile, several key articles you mentioned have been included in the reference list. In the ”Related Work” section, targeted reviews have been made on the core viewpoints and research methods of these articles, fully demonstrating the reference and response to important research achievements in the field and avoiding the problem of relying on a single literature.

⋮

Reviewer Comment

> 3.All detection algorithms rely on underlying mathematical principles. The absence of a mathematical framework in your paper renders your method insufficient. Without using a mathematical equations, the methodology section is still weak and unclear.

Response

We deeply recognize the significance of mathematical expression in clearly presenting the logic of methods. We have added a dedicated section in the ”Methodology” section of the revised draft, systematically supplementing the mathematical derivation and formula definition of the core method of this study - specifically including the mathematical expression of the CPE function and the complete formula of the loss function constructed based on this function At the same time, the explanations of the meanings of each symbol in the formula have been supplemented.

Reviewer Comment

> 4.There is a lack of description of Fig. 1 and Fig. 2. Please add a brief description-a short explanation of what the figure illustrates.

Response

Based on your suggestion, we have supplemented the brief descriptions of Figures 1 and 2 in the original text as follows: Figure 1 mainly introduces the basic structure and training process of the FSCE network: structurally, it clearly defines the core modules and data flow direction; in the training process, it explains the complete procedure and the key training strategies in the feal-shot scenario, which helps to understand the network operation mechanism. Figure 2 focuses on elaborating the improvement contents and reasons of FSCE in this paper: marking the optimization

module, explaining the original intention of improvement one by one in combination with the deficiencies of the original network, and clearly demonstrating the innovation points and rationality of the work in this paper.

Reviewer Comment

> 5.There are a lot of different classifier methods. The paper includes only a Softmax classifier to design an ensemble model. Why? Did you try other classifiers when conducting a set of your experiments or were you depending on previous studies? Authors should discuss this point in the discussion section to be clarified in their manuscript so that their model can actually be supported.

Response

The FSCE algorithm is based on the framework of Faster R-CNN. In the classic structure of Faster R-CNN, the classification module at the end of its detection process adopts the traditional Softmax classifier. This paper takes FSCE as the research benchmark and makes targeted improvements to its classification module - replacing the traditional Softmax classifier in the original architecture with a cosine Softmax classifier. To verify the improvement effect, comparative experiments were conducted on two public datasets. The results showed that the improved algorithm achieved significant improvements in the key evaluation metric AP (Average Precision). Specifically, the AP values on the two datasets increased by 2.1 and 1.56 respectively. It fully proves the effectiveness of the improved scheme of this classifier.

---

6. Reviewer Comment

> 6.The quality of a manuscript's results section is significantly improved by the use of well-designed, clear, and appropriate figures and tables that effectively present complex data, summarize key findings, and help readers visualize trends and significant information without unnecessary text. However, in reviewing your manuscript, I noticed that your manuscript's results just included tables without figures, which may limit the clarity of your findings and the chance of acceptance of the manuscript for publication.

Response

To more comprehensively evaluate the performance of the proposed model, we conducted supplementary experiments on the FLIR dataset: calculating the mAP (i.e., the average of all category AP values) of different comparison models, and drawing the corresponding curve graph based on this metric to visually present the performance differences of each model on this dataset, providing more sufficient support for the model performance evaluation.

---

7. Reviewer Comment

In reviewing the bibliography, I noticed there is a repetition of the same reference [15, 17] in the bibliography. It should be corrected.

Response

Thank you very much for your meticulous review and for pointing out the issue of duplicate references. We have checked the reference list and confirmed that the reason for the repetition of reference [15,17] is that there is an error in the citation information of reference [17]. At present, we have corrected reference [17] to the accurate content to ensure that it does not duplicate reference [15]. In addition, we have conducted a comprehensive review of all the references in the full text to avoid similar errors and ensure the completeness and accuracy of the reference list.

---

8. Reviewer Comment

In the abstract, the Future work is unclear. It needs more explanation to guide the reader on the next steps.

Response

In response to this issue, we have provided a detailed elaboration on future work in the summary section of the article: First, deploy edge devices, complete hardware selection and debugging, and set up the hardware environment; The second is to optimize the algorithm and achieve lightweighting through pruning and other means; The third is to enhance the model's accuracy, iterate on existing methods and try new solutions; The fourth is to introduce large language models to replace traditional neural networks and innovate the technology.
