# Supplementary material for: A novel few-shot object detection framework for multi-scene driving based on contrastive proposal encoding
Source: PLoS One. 2025 Dec 30;20(12):e0337541. doi: 10.1371/journal.pone.0337541 (PMC12752960; doi:10.1371/journal.pone.0337541)
Supplement: Attachment — Submitted filename: PLOSONE Reviewer-2025.docx [file pone.0337541.s001.docx]

**A Novel Few-Shot Object Detection Framework for Multi-Scene Driving Based on Contrastive Proposal Encoding**

The manuscript entitled “A Novel Few-Shot Object Detection Framework for Multi-Scene Driving Based on Contrastive Proposal Encoding”. Authors enhanced a few-shot object detection algorithm depended on Contrastive Proposal Encoding in order to improve both the accuracy and generalization capability of the model. They employed two types of datasets to evaluate the detection performance. However, the manuscript has several critical weaknesses. Below are the major comments for revision:

1. Before starting the methodology, it would be better to provide summary of related works including Author, year, dataset, models, best model, feature selections techniques, accuracy, and processing time ….et. because the related works did not clearly indicate the research gaps.
2. Before starting the methodology, it would be better to provide a summary of related works, including author, year, dataset, models, best model, feature selection techniques, accuracy, and processing time, as the related works did not clearly indicate the research gaps. Additionally, your proposal is based on ref.[15] largely. However, the reference [15] is often not the dominant one for improving your method. Therefore, to enhance the manuscript, consider incorporating more recent advances and relevant research works.
3. All detection algorithms rely on underlying mathematical principles. The absence of a mathematical framework in your paper renders your method insufficient. Without using a mathematical equations, the methodology section is still weak and unclear.
4. There is a lack of description of Fig. 1 and Fig. 2. Please add a brief description- a short explanation of what the figure illustrates.
5. There are a lot of different classifier methods. The paper includes only a Softmax classifier to design an ensemble model. Why? Did you try other classifiers when conducting a set of your experiments or were you depending on previous studies? Authors should discuss this point in the discussion section to be clarified in their manuscript so that their model can actually be supported.
6. The quality of a manuscript's results section is significantly improved by the use of well-designed, clear, and appropriate figures and tables that effectively present complex data, summarize key findings, and help readers visualize trends and significant information without unnecessary text. However, in reviewing your manuscript, I noticed that your manuscript's results just included tables without figures, which may limit the clarity of your findings and the chance of acceptance of the manuscript for publication.
7. In reviewing the bibliography, I noticed there is a repetition of the same reference [15, 17] in the bibliography. It should be corrected.
8. In the abstract, the Future work is unclear. It needs more explanation to guide the reader on the next steps.
